# Application and research progress of machine learning in the diagnosis and treatment of neurodevelopmental disorders in children

**DOI:** 10.3389/fpsyt.2022.960672

**Published:** 2022-08-24

**Authors:** Chao Song, Zhong-Quan Jiang, Dong Liu, Ling-Ling Wu

**Affiliations:** ^1^Department of Developmental and Behavioral Pediatrics, The Children's Hospital, Zhejiang University School of Medicine, National Clinical Research Centre for Child Health, Hangzhou, China; ^2^School of Public Health, Lanzhou University, Lanzhou, China; ^3^Department of Neonatology, Shenzhen People's Hospital, Shenzhen, China

**Keywords:** artificial intelligence, machine learning, child, neurodevelopmental disorder, diagnosis, treatment

## Abstract

The prevalence of neurodevelopment disorders (NDDs) among children has been on the rise. This has affected the health and social life of children. This condition has also imposed a huge economic burden on families and health care systems. Currently, it is difficult to perform early diagnosis of NDDs, which results in delayed intervention. For this reason, patients with NDDs have a prognosis. In recent years, machine learning (ML) technology, which integrates artificial intelligence technology and medicine, has been applied in the early detection and prediction of diseases based on data mining. This paper reviews the progress made in the application of ML in the diagnosis and treatment of NDDs in children based on supervised and unsupervised learning tools. The data reviewed here provide new perspectives on early diagnosis and treatment of NDDs.

## Introduction

Neurodevelopmental disorders (NDDs) including autism spectrum disorder (ASD), attention deficit hyperactivity disorder (ADHD), intellectual disability (ID), and learning disability (LD) are a class of diseases that affect brain development and function. These disorders occur during early development and affect the cognitive and emotional development of children (1–3). Evidence shows that burden of NDDs in children is becoming a global challenge, affecting about 3% of children worldwide (4). The incidence of NDDs has been on the rise globally. In ASD, the 2020 monitoring network report by the Centers for Disease Control and Prevention revealed that the prevalence of ASD among 8-year-old children was 1.68%, representing a 10% increase compared with 2018 (5). In 2021, a surveillance report showed that the prevalence of ASD had risen to 2.27% or 1 in every 44 children (6). Moreover, several meta-analyses have reported varying global prevalence rates. For instance, the prevalence of ADHD in children was 7.2% (7), that of ID was 1–3% (8), whereas that of LD was 3–8% (9, 10). Of note, NDDs affect the health and social functioning of children, as well as imposes huge economic burden on families (11, 12).

Studies have shown that NDDs is mainly caused by genetic and environmental factors. However, the pathogenesis of NDDs, represented by ASD/ADHD, is unclear and there are no accurate biomarkers of this disorders (13). Currently, early diagnosis of NDDs is difficult due to the high heterogeneity of its phenotypes and etiological factors (14). This results in delayed intervention. Therefore, there is an urgent need to develop strategies for improving early detection and prediction of NDDs. In clinical practice, NDDs are mainly diagnosed based on behavioral symptoms of children and information provided by caregivers (2, 15). This calls for development of standardized diagnostic neuropsychological testing tools for this condition. Moreover, diagnosis based on behavioral symptoms is not accurate because it dependents on the pediatricians' experience and observation time. Currently, only about 8% of pediatric providers have the skills to diagnose NDDs (16). There are differences in the reliability and validity of standardized test tools for NDDs, but such tools cannot be easily obtained, due to geographical or cultural reasons (17). Currently, no testing tool or scale can directly diagnose NDDs. Even the available Autism Diagnostic Observation Scale and Autism Diagnostic Interview-Revised guidelines regarded as the “gold standard” for ASD diagnosis may lead to misdiagnosis (18).

Considering the inability of single scales, tools or indicators to accurately diagnose or predict NDDs, it has been proposed that objective index data (e.g., socio demographic information, EEG, skull imaging) should be combined to improve the diagnosis or prediction of NDDs. Machine learning (ML) has been found to offer good predictive performance on the occurrence of NDDs (19). Several ML methods such as, supervised, unsupervised, semi-supervised, and reinforcement learning, have been used in the diagnosis and treatment of NDDs (20–22). Semi supervised learning and reinforcement learning are rarely used in the field of NDDs. Semi-supervised learning and reinforcement learning are rarely used in the field of NDD with its unique data processing advantages, ML can facilitate the early identification and early diagnosis of NDD. Reviewing the progress of ML in the field of NDD is a reflection of the cross-fertilization of medicine and engineering, which helps to expand the boundaries of ML applications and deepen the understanding of NDD among medical professionals. Therefore, this paper focuses on the application of supervised and unsupervised learning in NDD to provide a scientific basis for improving the quality of life of NDD patients.

## Supervised learning

Supervised learning can be applied in early detection, prediction of NDDs, and identification of risk factors. Regression analysis, decision tree, support vector machine, and artificial neural network are the commonly used supervised ML methods.

### Regression analysis

Regression analysis is the most basic and widely utilized ML model. Linear regression, logistic regression, and regularized regression are interpretable and are extensively. For instance, Wang et al. adopted multivariate binary logistic regression analysis to identify factors associated with ASD. They found that gender, living area, age, and education level are contributing factors contributing to ASD occurrence (23). Tourette syndrome (TS) is the most common neurodevelopmental movement disorder (2). Elsewhere, Burd et al. used binary logistic regression analysis to develop a regression model for evaluating factors contributing to TS. They found that being male, without a family history of TS, and high number of comorbidities influence the occurrence of TS (24). Bertoncelli et al. established a binary logistic regression analysis model comprising 91 adolescents with cerebral palsy for predicting cerebral palsy in children and the associated risk factors. The average accuracy, specificity and sensitivity of the model were 78%. It also suggested that poor motor skills, epilepsy and cerebral palsy were related risk factors. This implies that a prediction model based on binary logistics can effectively identify children with cerebral palsy (25).

There are a lot of influential factors in NDDs, which inevitably leads to collinearity problems. If these factors are not controlled and filtered, they affect the model performance and even lead to production of misleading results. To address this problem, regularization technology has been proposed. In the European multicenter children's TS study (EMTICS), 187 first-degree relatives of TS children aged between 3 and 10 were followed up for 7 years. Subsequently, a lasso logistic regression prediction model for Tourette was established. The interpretation of this method were relatively simple and its prediction accuracy was good (26), indicating the extensive use of regression analysis in the field of NDDs.

### Decision tree

The decision tree was first proposed in 1986 (27). It possesses tree classifier classification properties and can produce interpretable and accurate results without parameter assumptions. Iterative dichotomiser 3 (ID3), classification and regression tree (CART) are the most widely used to generate medical decision rules for NDDs. Mohamma et al. used features such as, child behavior, neuropsychology, and electrophysiological markers to build models. They then constructed an early childhood predictive model for ADHD using the classic ID3 algorithm. They reported that the decision tree model yielded excellent classification accuracy (100%). Also, subtypes of ADHD can be distinguished by key nodes in decision-making rules such as behavioral, neuropsychiatric and electrophysiological parameters (28). New algorithms based on classical decision tree algorithms, including the ones using alternate decision trees, multi-class alternate decision trees, have been used to construct models based on genomic and magnetic resonance data. It has been found that the decision tree outperforms other ML models. Consequently, rs878960 in GABRB3 (gamma-aminobutyric acid A receptor, beta 3) has been selected by all tree-based models (29). In practical application, the decision tree is prone to overfitting. Effective sampling methods and pruning methods should be developed to solve the problem of overfitting. CART, which is extensively used, utilizes a cost complexity pruning algorithm. Previously, the predictive significance of birth weight, term infants, and Apgar score in ADHD was explored. A total of 132 boys diagnosed with ADHD and 146 typical developmental boys in the control group. The decision tree model constructed using the CART algorithm revealed that the Apgar score used to reflect the degree of neonatal asphyxia had the highest predictive value, whereas a low Apgar score was among the most critical risk factors in the perinatal period of ADHD children, suggesting that perinatal asphyxia may be related to later occurrence of NDDs symptoms. Therefore, application of complexity pruning algorithm for post pruning improves the prediction accuracy of the decision tree (30).

### Support vector machines

Previously, Cortes et al. proposed a linear classifier model which had the largest spacing in feature space and a support vector machine (SVM). The model can solve a separation hyperplane that correctly divides the training dataset with the largest geometric intervals (31). SVM has good performance on small sample implementations. Notably, linear kernel functions, polynomial kernel functions, sigmoid, radial basis function kernels are frequently utilized kernel functions. For instance, Conti et al. used retrospective cohort data from 68 children aged 34–74 months from the head of MRI to construct an early differential diagnostic model of ASD and Childhood Apraxia of Speech (CAS) of linear nuclear function SVM. It was found that the linear kernel function SVM model effectively achieved early differential diagnosis and individualized intervention of ASD and CAS (32). Similarly, Agastinose Ronicko et al. used Gaussian kernel SVM, random forest, and convolutional neural network to construct a predictive model based on Resting-state functional Magnetic Resonance Imaging (Rs-fMRI) data for early diagnosis and treatment of ASD. They found that compared with other machine learning mentioned above, Gaussian kernel SVM has stronger performance in early diagnosis and treatment of ASD (33). To improve the performance of individual SVM classifiers, Bi et al. constructed an ensemble SVM model by integrating Rs-fMRI data from 46 normal children and 61 children with ASD. The proposed ensemble SVM model showed good classification performance based on all features, implying that the ensemble SVM method can be used as an auxiliary diagnosis of ASD (34). Objective imaging data obtained by Rs-fMRI technology is more effective for the diagnosis of ASD compared with behavioral observation. SVM has excellent performance in the above imaging data and small samples.

### Artificial neural network

An artificial neural network (ANN) is a complex network structure formed by interconnection of numerous processing units. It is a form of abstraction, simplification, and simulation of the structure and operation mechanism of the human brain. ANN can perform simulations, image recognition, and prediction functions. In an investigation aimed at evaluating the relationship between athletic capacity and other clinical features of ASD, Fulceri et al. performed exploratory analysis via ANN. Poor motor performance is a common clinical feature in preschoolers with ASD, associated with repetitive stereotyped behaviors and weak language skills (35). Single-layer neural networks cannot solve the XOR problem in the context of artificial neural networks. In contrast, two-layer neural networks can resolve this problem. At the same time, it demonstrates a strong non-linear classification effect. Rumelhar et al. proposed the Back Propagation (BP) algorithm in 1986 (36). BP solves the complex computational quantity problem required by two-layer neural networks and the computational problem of multilayer perceptron (MLP). The concept of implicit layer was introduced to act as a kernel function of an SVM that maps sample spaces to high-dimensional linear separable spaces. Moreover, Hossain et al. analyzed demographic data, clinical indicators, and imaging data to identify ASD features and construct the MLP classifier model to improve the accuracy of automated diagnosis of children with ASD. It was observed that the MLP outperformed all other benchmark classification models, achieving a 100% accuracy with the lowest number of attributes in the toddler, child, adolescent, and adult datasets (37).

With the development of computer technology, the number of layers of neural network is increasing, and the problem of local optimal solution is becoming more and more prominent. The “convolutional kernel” is an intermediary, model which ensures that the original position relationship is preserved after an image is convoluted, thereby limiting the risk of falling into a locally optimal solution. Therefore, several convolutional neural networks (CNNs) have been proposed. Thomas et al. trained 3D-CNNs on an open ASD dataset to distinguish ASD using Rs-fMRI images and constructed a CNN-based ASD recognition model. Results showed that 3D-CNN had better distinguishing effect. Moreover, its performance exceeded that of the SVM model. However, valuable information cannot be extracted from time series in 3D-CNNs (38). Scientists have developed a long and short-term memory model (LSTM) to solve the disappearance of gradients in time. This model fulfills the time memory function by switching the gate and preventing the gradient from disappearing. Vikas et al. developed CNN, LSTM, and MLP (based on DSM-V) models for accurate diagnosis and assessment of severity of individuals with ASD. Comparative analysis revealed that LSTM functions better in the diagnosis of ASD unlike other neural network algorithms (e.g., CNN, MLP). This suggests that AI algorithms can improve the diagnosis of ASD (39). DSM-V is the most widely used diagnostic criteria for NDDs worldwide. The combination of DSM-V and ML not only enriches the connotation of DSM-V, but also proves that ML is suitable for the diagnosis and treatment of NDDs.

### Ensemble learning

Ensemble learning accomplishes learning tasks by constructing and integrating multiple weak learners. Common ensemble learning methods include boosting, bagging, and stacking (40–42). AdaBoost is an efficient boosting algorithm that allows weak learning algorithms with approximate random accuracy to be strong learning algorithms (43). PU Putra et al. explored responses and gaze performance of children during Go/No-Go missions. Based on the AdaBoost algorithm, the eye tracker was used to track the gaze data of children and construct a distinguishing model for ASD. As a result, the accuracy rate of AdaBoost's algorithm predicting ASD reached 88.60%, which has an application value (44). The collected the gaze data was huge and complex, and it was difficult to analyze such data with traditional statistical methods, and can only be processed by ML.

Of note, the Bagging algorithm is a parallel integration strategy that differs from Boosting. Bagging insights are applied to decision trees to obtain random forest models, further improving the predictive performance of the decision tree model (45). Feczko E et al. utilized Rs-fMRI brain connection data from 47 children with ASD and 58 healthy children to construct a random forest model to distinguish ASD. The findings showed a prediction accuracy of the random forest model of 72.71%, a specificity of 80.74%, and a sensitivity of 63.15%. Besides, unique behavioral characteristics of 3 ASD and 4 subsets of normal children were simultaneously revealed, showing that the random forest model performs effectively with extremely high value in the interpretation of features (46). In an exploratory analysis, random forests are extensively used for favorable robustness. Gao et al. sampled feces from 49 tic children and 50 healthy children for intestinal microbiome analysis to investigate the intestinal microbial features in tic patients and the effects of dopamine receptor antagonist (DRA) drugs on the composition and metabolic function of the intestinal microbiota. A random forest model was constructed to predict tic. The results showed that the model had an AUC of 0.884. Moreover, a significant correlation was noted between the severity of tic symptoms and abundance of multiple bacteria as well as the metabolic function of the gut microbiota (47).

Based on boosting and bagging, a stacking technique using different models for integration has emerged (48), however, literature related to NDDs is few; therefore, the application value warrants further investigations.

## Unsupervised learning

Unsupervised learning aims to train a model to learn the data structure, then provide valuable information about a new sample. The most significant distinction between unsupervised and supervised learning is whether the data contains learning labels or not. The most common scenarios for unsupervised learning include association rules, clustering, and dimensionality reduction.

### Association rule

Association rule use metrics to differentiate between strong rules existing in a database. The most common algorithm that uses this rule is the Apriori algorithm (49). Kim et al. applied the Apriori algorithm to extract ADHD comorbidities in Korean national health insurance data. Mood/affective disorders were the most common comorbidities of ADHD. Based on the outcomes of the association rules, 9 association rules were generated, providing a reference for subsequent research on ADHD (50). Many comorbidities are among the characteristics of NDDs. Such comorbidities can be used in the differential diagnosis of NDDs. ML provides a new path for early identification of comorbidities in NDDs, and it can also help to formulate more comprehensive intervention plans to improve outcomes in children with NDDs. Tai et al. also used the Apriori algorithm to evaluate the comorbid network of children with ADHD. Consequently, the risk of comorbidity between ADHD and psychosis was significantly higher than that with other physical diseases (51). Similarly, association rules can also be used in diagnostic models. For instance, Ucuz et al. investigated the effects of temperament and character traits on ADHD diagnosis. A diagnostic model of ADHD was established based on the classification-based association rules method. Data were collected from 36 children with ADHD and 39 healthy children. The results showed that the diagnostic model based on association rules had good discrimination performance, and temperament personality characteristics can be used for the clinical diagnosis of ADHD (52).

### Clustering

Clustering involves dividing a dataset into different classes or clusters based on a set of criteria, to maximize the similarity of data objects within a cluster, while minimizing the difference between data objects that are not in the same cluster. K-means is the most conventional clustering method; it classifies points in n-dimensional space based on the degree of Euclidean distance. Vargason et al. explored ASD complications and the ASD subtypes in the United States between 2000 and 2015 using a database with 3,278 insured children with ASD and 279,693 children with ASD. K-means algorithm was used to identify three subgroups of children with ASD. Meanwhile, there was a strong association between developmental delay and ASD in comorbidities, followed by gastrointestinal problems and immune imbalances. Suggestive clustering results potentially help in screening children with ASD for comorbidities and understanding ASD subgroups (53). In practice, the k-means algorithm has several limitations such as, s specifying the initial number of class clusters and easy overfitting, without obtaining the cluster tree. Therefore, researchers often utilize hierarchical clustering and Gaussian mixed models. For instance, Stevens et al. used hierarchical clustering and Gaussian mixed models to cluster the behavioral phenotypes of ASD and therapeutic outcomes of different phenotypes. This approach provided a scientific reference for personalized interventions (54).

### Dimensionality reduction

Clinical data are complex comprising redundant data, which improves the accuracy of model recognition by minimizing dimensionality. At the same time, it also highlights the important structure of data. Of note, principal component analysis (PCA) is the most commonly used linear dimensionality reduction method. The features of origin data points are preserved while data dimensions are reduced (55). For example, N Mashal et al. performed principal component analyses on 37 ASD, 20 LD, and 21 normal children to address the interrelationships between various tests in each group. The results revealed no dichotomy between visual and verbal metaphors in healthy children. Instead, metaphors were categorized as per their familiarity. In the LD group, visual metaphors were independently categorized as linguistic metaphors. The verbal metaphorical understanding of the ASD group was similar to that of the LD group (56). Additionally, when processing and analyzing a complex image and audio data, Ousts et al. applied PCA technology to minimize data dimensionality, thereby stabilizing the subsequent modeling (57). This suggests that dimensionality reduction methods including PCA should be appropriately used to increase the model stability in processing complex data.

## Discussion

In summary, supervised algorithms can be used to develop models for NDDs diagnosis and prediction. Unsupervised algorithms can be applied in exploratory research or optimization of data structures to identify associations between NDDs or key risk factors of a single disorder. Supervised algorithms have varied applicability to different NDDs data structures due to their different algorithm structures. Artificial intelligence has been shown to have good performance on imaging data. For large data samples, ensemble learning often shows fast computing power and performance. In few-shot training, SVM performs well (Table 1). At present, most of the NDDs diagnosis and prediction models built based on ML do not follow the standard *The Transparent Reporting of a multivariable prediction model for Individual Prognosis Or Diagnosis* (TRIPOD) clinical prediction model reporting specifications (68), such as the lack of processing of missing values and outliers in the reporting process, and the failure to report the threshold of the model. This makes the model difficult to reproduce. For model evaluation, multi-dimensional evaluation (e.g., discrimination, calibration, clinical usefulness, etc.) is rarely used, and it is difficult to effectively screen out a model that is truly suitable for samples only from a single discrimination dimension. In terms of model verification, most studies only evaluate the performance of the model on the current sample from the perspective of internal verification, and there is a certain risk of overfitting. Most studies lack the consideration of model generalization ability on external validation based on external data.

**Table 1:** Advantages and disadvantages of supervised learning and unsupervised learning methods.

	**Advantages**	**Disadvantages**
**Supervised learning**		
Regression analysis (25, 26, 58–61)	1. Simple modeling and strong interpretability 2. Independent of parameter adjustment, the same model and data can usually calculate the unique result	1. Sensitive to missing and abnormal values 2. Logistic regression is difficult to deal with nonlinear problems 3. It is difficult to deal with multicollinearity problems 4. There is risk of over fitting 5. Sensitive to unbalanced data
Decision tree (28–30, 62–66)	1. The model is highly interpretable 2. The model can be described graphically 3. Insensitive to continuous and discrete data 4. It can process multi classification data 5. Insensitive to missing and abnormal values 6. It does not depend on background knowledge and can be modeled directly	1. The decision tree without pruning has the risk of over fitting 2. Sensitive to unbalanced data 3. The performance of the model is generally weaker than that of ensemble learning and regression analysis
SVM (31)	1. It has complete theoretical support, especially suitable for small sample research 2. The computational complexity depends on the support vector, which avoids the disaster of dimensionality to a certain extent 3. A few support vectors determine the final result, reducing the impact of miscellaneous samples on the model 4. Insensitive to outliers	1. It is difficult to train on big data samples 2. It is difficult to solve the multi classification problem 3. The model depends on parameter selection
ANN (38, 39, 67)	1. Strong nonlinear mapping ability 2. Have the ability to associate input information, self-learning and adaptive ability 3. Have a strong ability to distinguish training samples 4. Convolution algorithm can recognize imaging data well	1. The model has the risk of over fitting 2. Large amount of model calculation 3. Complex imaging data analysis
Ensemble learning (40–42, 44, 48)	1. The performance of the model is improved to a certain extent compared with the weak classifier 2. Insensitive to outliers 3. High performance on large samples 4. It can deal with nonlinear problems 5. Random forest is not sensitive to unbalanced data 6. Little possibility of over fitting	1. The model is difficult to explain, and there is a black box problem 2. Normalization is required 3. Some models are sensitive to missing values
**Unsupervised learning**		
Association rule (49, 50)	1. The algorithm principle is simple and easy to implement 2. It is not restricted by dependent variables, and the association between data can be found in big data	1. There are many output rules and a lot of useless information
Clustering (53, 54, 61)	1. The principle is relatively simple, the implementation is also very easy, and the convergence speed is fast 2. Be able to handle big data problems 3. Strong interpretability	1. The model is sensitive to outliers 2. The model is sensitive to unbalanced data 3. Local optimal solutions are often obtained
Dimensionality reduction (55, 56)	The model is fast, simple and effective	Poor interpretability of the model

Nowadays, several studies have attempted to develop ML clinical diagnostic evaluation tools for NDDs. For example, the ASD diagnosis and assessment tool based on questionnaire data was recently developed by *De novo*. This tool was approved by the Federal Drug Administration for pre-marketing review, which is the first successful application of ML in the early diagnosis and early screening of NDDs (69). More companies, such as ALSOLIFE, are attempting to develop ASD auxiliary diagnostic tools based on ML from imaging data. However, in the field of NDDs research, ML models have numerous limitations. For example, the heterogeneity of ASD in phenotype and pathological mechanism leads to inconsistent performance and result interpretation of ML models on different training samples (14), and it is impossible to obtain a ML model suitable for the entire ASD population. In addition, the training of supervised ML models relies on existing samples, and for NDDs, there is no database of existing samples. Currently, numerous diagnostic models based on clinical imaging data (32, 33, 38), have been reported such as Rs-fMRI and EEG. However, the cost of obtaining these data is high and this imposes a huge economic burden on the patient's family. Even if the ML model has excellent performance on these data, its application in the diagnosis of NDDs is challenging.

There are several limitations of this review article. First of all, this paper focuses on the applicability of ML in the diagnosis and treatment of NDDs, so the subject content of the cited literature is reviewed. Some literature did not present the data in full, so it was impossible to strictly checked the data quality of the cited literature. Second, NDDs are a class of diseases, and the pathogenesis, clinical manifestations, treatment options and prognosis of each disease in NDDs are different. At the same time, the obtained data also have various degrees of difference, and the analysis of different diseases still needs to be combined with the characteristics of the disease data. Currently, there is no single ML method or model that works for all data types. At present, the application of ML in a certain NDDs has been reviewed, and this kind of research is also very meaningful. Finally, since NDDs are a current research hotspot, some of the views in this paper may become incomplete as ML applications in the field further increase.

In conclusion, the benefits of ML in the diagnosis and intervention of NDDs are taking shape with its excellent performance and interpretability. Integration of medical big data and ML may be an effective strategy to guide the diagnosis, intervention, and prognosis of NDDs. Collecting clinical big data of NDDs and constructing models scientifically are the work that can be set out now.

## Author contributions

CS conceived the study and critically revised the article. Z-QJ, DL, and L-LW performed literature search and drafted the manuscript. All authors contributed to the study and approved the final version to be submitted.

## Funding

This study was supported by the Zhejiang Nature Science Foundation of China (LGF20H090015).

## Conflict of interest

The authors declare that the research was conducted in the absence of any commercial or financial relationships that could be construed as a potential conflict of interest.

## Publisher's note

All claims expressed in this article are solely those of the authors and do not necessarily represent those of their affiliated organizations, or those of the publisher, the editors and the reviewers. Any product that may be evaluated in this article, or claim that may be made by its manufacturer, is not guaranteed or endorsed by the publisher.

## Abbreviations

ADHD: Attention deficit hyperactivity disorder
ASD: Autism spectrum disorder
ANN: Artificial neural network
BP: Back Propagation
CART: Classification and regression tree
CNN: Convolutional neural network
DRA: Dopamine receptor antagonist
ID: Intellectual disability
ID3: Iterative dichotomiser 3
LD: Learning disability
LSTM: Long and short-term memory model
ML: Machine learning
MLP: Multilayer perceptron
NDD: Neurodevelopment disorder
PCA: Principal component analysis
Rs-fMRI: Resting-state functional Magnetic Resonance Imaging
SVM: Support vector machines
TS: Tourette syndrome.
